# Blistering Bulbs: The Hidden Dangers of Garlic as a Home Remedy

**DOI:** 10.7759/cureus.100429

**Published:** 2025-12-30

**Authors:** Riley Bunstine, Christopher M Lloyd

**Affiliations:** 1 Emergency Medicine, OhioHealth Doctors Hospital/Ohio University Heritage College of Osteopathic Medicine, Columbus, USA

**Keywords:** burns, emergency medicine, garlic, home remedies, skin lesions

## Abstract

We report a case of a 46-year-old male who developed a second-degree chemical burn after application of crushed raw garlic to his left wrist. When crushed, garlic activates the enzyme alliinase that converts alliin into allicin. In controlled concentrations, allicin has been shown to have a wide array of therapeutic benefits; however, its use as an unregulated home remedy carries a significant risk of toxicity. This patient applied crushed garlic to his left wrist and left this poultice in place for five hours, hoping to ease wrist pain from a previous car accident. Upon removal, he felt an intense burning sensation and noticed significant blistering. In the emergency department, two large intact bullae were noted with no interstitial fluid leakage. While garlic-induced chemical burns are a recognized entity, this case highlights an uncommon and more rapidly evolving presentation than those previously described. This case report highlights the importance of proper patient education regarding the potential risks of seemingly benign at-home remedies.

## Introduction

Garlic (*Allium sativum*) has been used for centuries in traditional medicine due to its extensive range of proposed health benefits, including cancer prevention, anti-inflammatory effects, antioxidant properties, and the management of cardiovascular, bone, and metabolic disorders [1-5]. These effects originate from the organosulfur compounds found in garlic, including allicin, diallyl sulfide, S-allyl-cysteine, diallyl disulfide, and diallyl trisulfide [1-3]. Each of these compounds has unique chemical properties, and garlic's composition varies depending on its preparation and treatment methods. 

When fresh garlic is peeled and then chopped or crushed, it activates the enzyme alliinase, which quickly converts alliin into allicin [2,3]. Allicin is a highly reactive sulfur compound with both therapeutic and cytotoxic effects that vary between microbes and mammals due to differences in vulnerability and mechanism of action. Allicin acts by inhibiting sulfhydryl-dependent enzymes, such as alcohol dehydrogenase, thioredoxin reductase, and RNA polymerase. Mammals are naturally better protected from these effects because they possess both glutathione and cysteine, which can neutralize allicin before sulfhydryl-dependent enzyme inhibition occurs. Microbes, on the other hand, have limited or absent glutathione and are more susceptible to the cytotoxic effects of allicin [6,7]. 

Owing to this protective effect in mammals, concentrations of 10 to 25 µM/mL are considered therapeutic, with benefits including improved cellular function, reduced tissue damage, decreased pro-inflammatory cytokines (IL-6, TNF-α), improved oxidative stress markers, and antimicrobial activity [2,7,8]. Allicin has also been shown to inhibit lymphangiogenesis, an important process in tumor metastasis, and it has been proposed for experimental use in cancer prevention [2,4,8]. 

At higher concentrations of 50 to 100 µM/mL, the increased glutathione in mammals is no longer sufficient to prevent the inhibition of sulfhydryl-dependent enzymes. At these concentrations, studies have shown a rapid loss of cell viability and severe, irreversible cytotoxicity [7,8]. These effects are evident when precision in dosing and application is not maintained. When raw garlic is applied topically, high allicin concentrations can cause skin irritation, blistering, and chemical burns due to its cytotoxic nature [9-13]. While this may sound concerning, the organosulfur components of garlic change over time, as allicin is volatile and can gradually degrade into other, less harmful organosulfur compounds [2,3]. This degradation does not account for the resolution of skin injury; however, as garlic ages, its composition shifts toward compounds with milder effects and greater therapeutic potential.

When marketed as a naturopathic remedy, garlic is typically aged commercially in an ethanol bath over several months to isolate beneficial sulfur compounds, which are then extracted. Aged garlic extracts have been shown to benefit the skin by lowering pro-inflammatory cytokines, such as IL-6 and TNF-alpha, while also reducing oxidative stress and boosting antioxidant capacity [1-3,5]. This has shown promise for oral supplements and topical creams that regulate the concentration of allicin and focus on utilizing aged garlic’s more stable bioactive compounds.

## Case presentation

A 46-year-old male presented to the emergency department (ED) for evaluation of a burn to his left wrist. This patient has a significant past medical history of insulin-dependent type 2 diabetes, with occasional visits for hypoglycemia. His current medication list includes insulin NPH, lisinopril, omeprazole, and cetirizine.

Two days prior, the patient was involved in a motor vehicle collision associated with a hyperglycemic episode. He was evaluated in the ED and subsequently discharged with no significant traumatic findings identified. He returned to the ED two days later with chemical burns sustained after applying garlic paste to his wrist. The patient reported residual pain following a motor vehicle collision and, in an attempt to alleviate this pain, crushed fresh garlic into a paste and applied it to his left wrist, securing it with a cotton elastic bandage for approximately five hours before removal. He denied any overlying skin breakdown or abrasions from the accident, and it was unknown if he mixed the garlic with any other substances prior to application. Once removed, he noted blistering and a burning sensation around the area.

**Figure 1:**
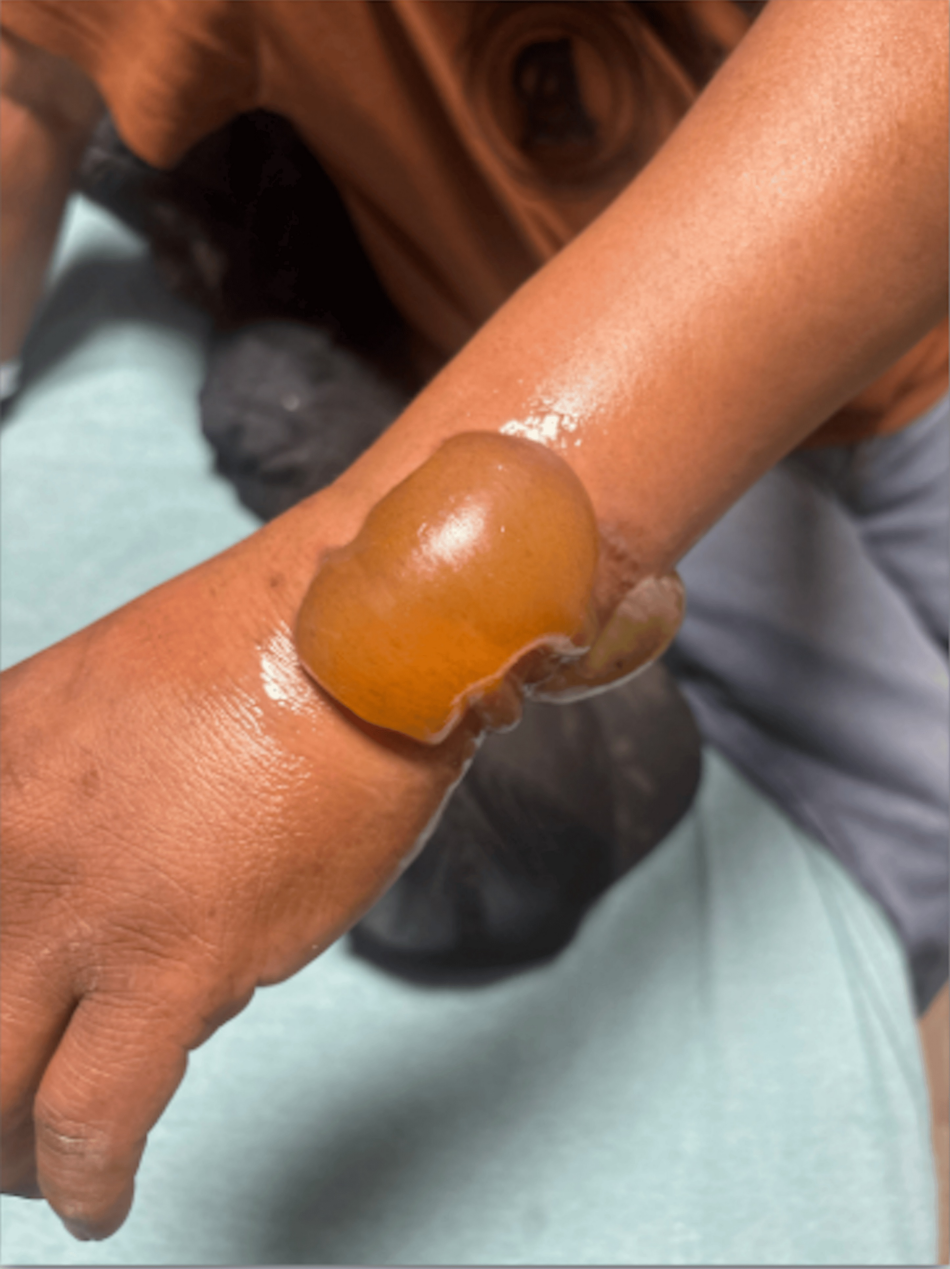
Left dorsal wrist showing second-degree burn

**Figure 2:**
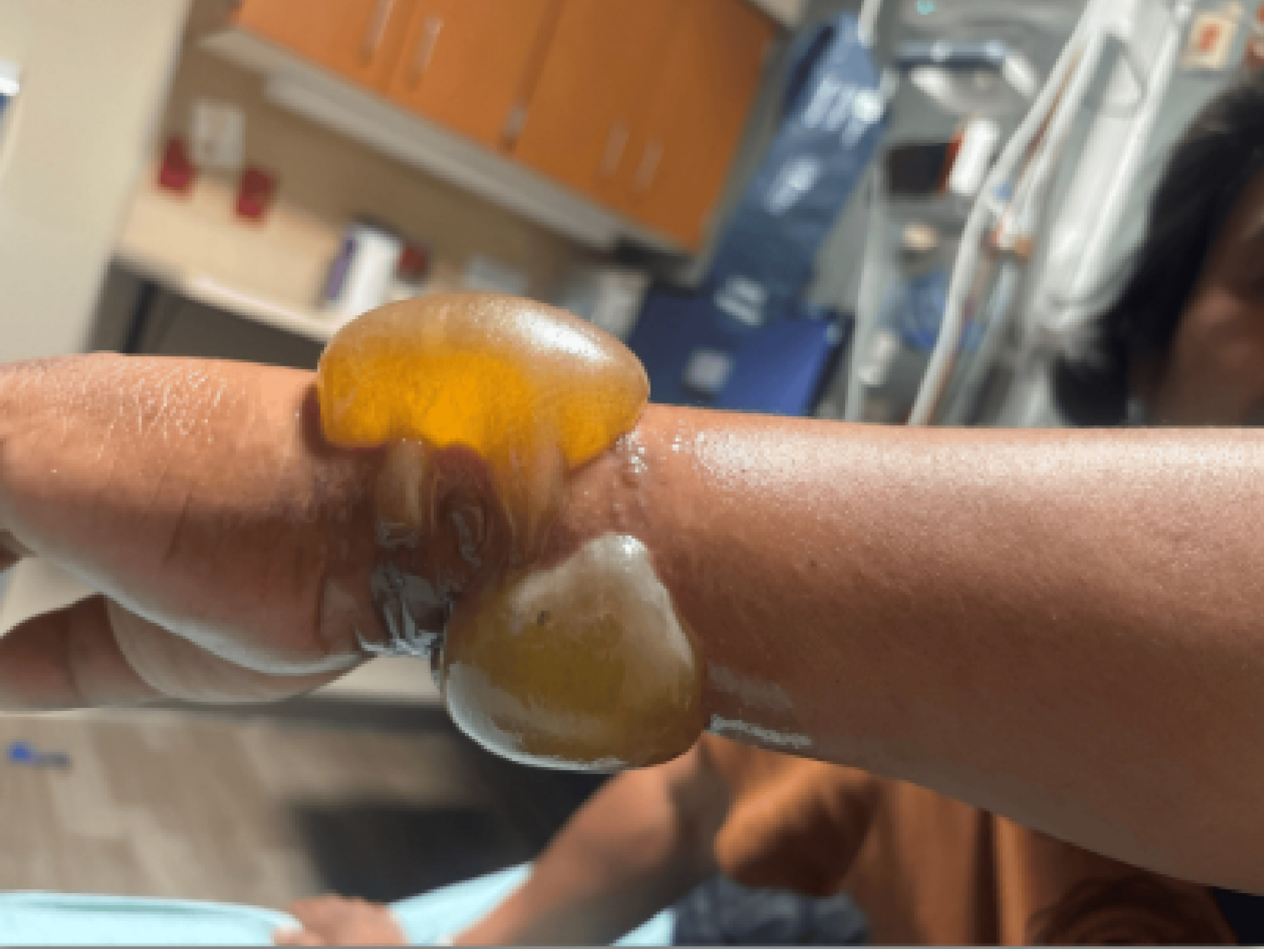
Left lateral wrist showing second-degree burn

**Figure 3:**
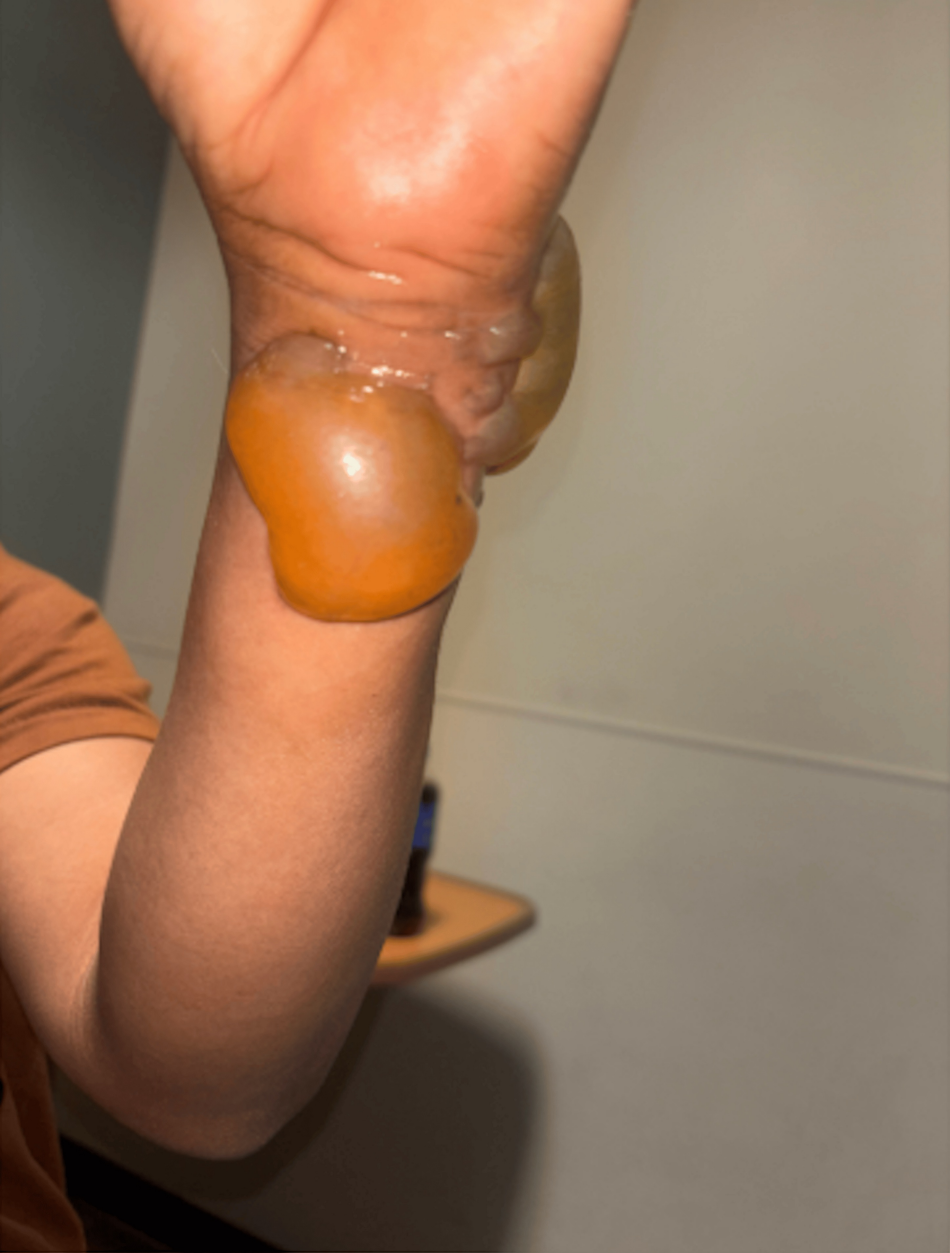
Left volar wrist showing second-degree burn

On examination, he was hypertensive (157/94 mmHg) and mildly tachycardic with a heart rate of 100 beats per minute, both of which were presumed secondary to pain. The remainder of his vitals were normal, and he was resting comfortably with no acute distress. Physical examination revealed significant blistering with bullae and surrounding erythema to the dorsal, ventral, and ulnar aspects of the left wrist consistent with second-degree burns (Figures 1-3).

**Figure 4:**
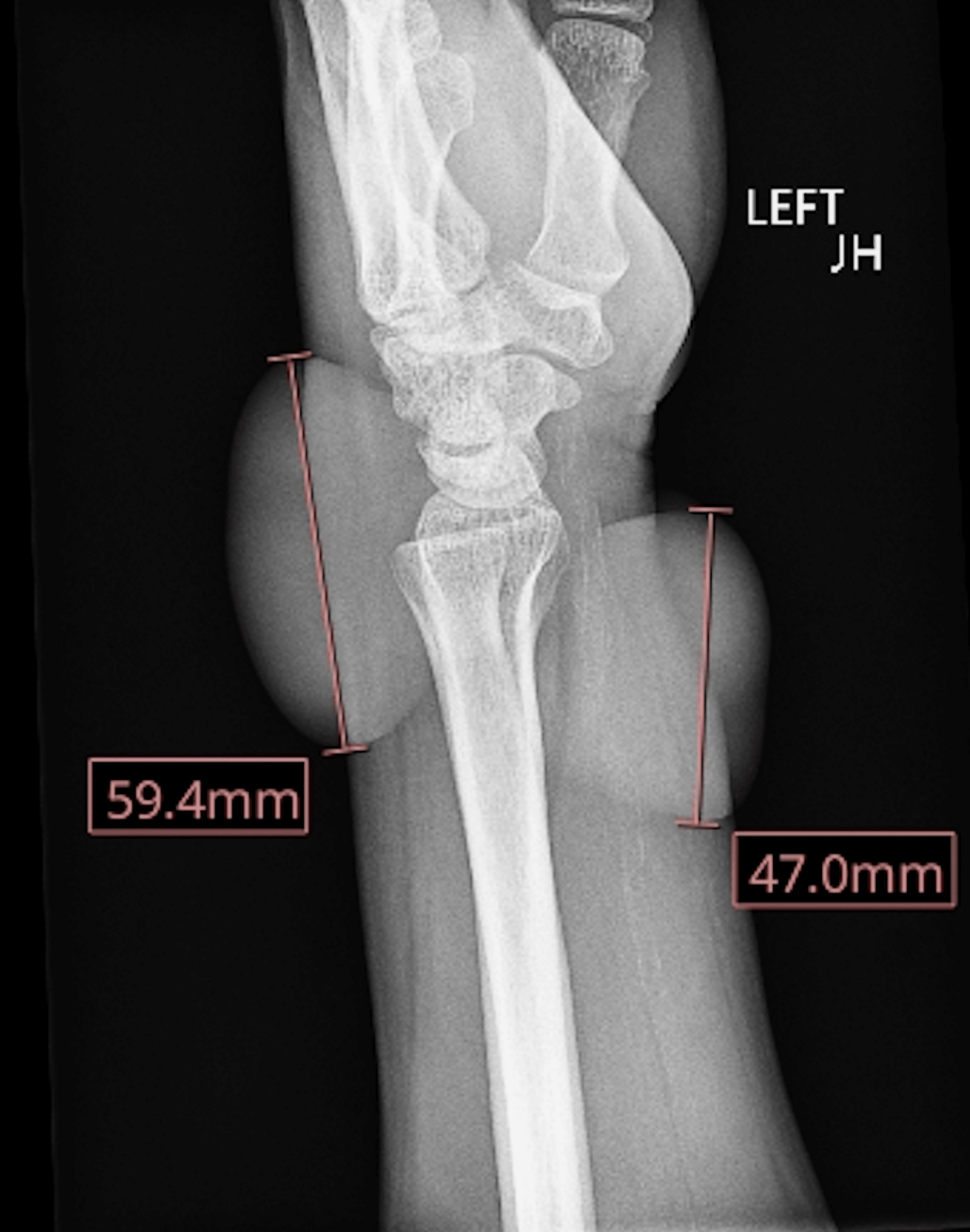
Lateral X-ray of the left wrist and wrist showing a lobulated lesion involving the dorsal aspect of the wrist and distal forearm consistent with the burn

There was tenderness to palpation and a limited range of motion of the wrist joint, and the neurovascular exam demonstrated normal sensory and motor strength. He was administered 5 mg of oxycodone and 325 mg of acetaminophen for pain, as well as tetanus prophylaxis. Radiographic imaging of the left hand and wrist was unremarkable, except for a lobulated lesion involving the dorsal aspect of the wrist and distal forearm consistent with the burn (Figure 4). 

The regional burn center was consulted primarily for follow-up care as the presentation was stable for outpatient management. Prior to discharge, the patient received topical bacitracin zinc ointment applied to the left wrist and covered with a non-adhesive dressing, as recommended by the burn center. The blisters were not popped or de-roofed in order to protect the underlying skin, decrease the incidence of infection, and avoid delaying wound healing. The patient was prescribed oxycodone 5 mg tablets to be taken as needed for pain. According to available electronic medical records, the patient did not attend his scheduled Burn Clinic follow-up and has not sought further medical care in the past three months. 

## Discussion

The initial differential diagnosis for this patient included a second-degree chemical burn, bullous pemphigoid, pemphigus vulgaris, and necrotizing fasciitis. These differentials were considered based on the presence of blistering, bullae, and surrounding erythema. The acute onset, localized distribution, and recent garlic application supported the diagnosis of a second-degree chemical burn.

Second-degree chemical burns following the application of raw crushed garlic, a phenomenon that, while documented, is rarely observed. Several case series have reported similar injuries resulting from the application of raw garlic. One review documented 39 total cases of garlic-induced burns between 1993 and 2021 [10]. This review included 21 female and 18 male patients, with ages ranging from 3 months to 80 years. Most burns were located on the legs (20), head (9), arms (6), thorax and abdomen (4). The use of raw garlic was frequently combined with additional bandages, dressings, or cloths similar to the application by this patient [10-12]. However, our case is notable for the burn’s unique presentation. Our patient presented with two large intact bullae with no interstitial fluid leakage. For comparison, multiple case reports, including a three upper limb garlic burns series, exhibited many small vesicobullous changes, loss of the outer skin barrier, and leakage of interstitial fluid [9,11-13]. Our patient also began to experience symptoms within 5 hours of exposure, whereas the above-mentioned case series continues to support that the average time required for burn development is 6 to 8 hours in children, and even longer in adults [10]. 

The patient’s atypical presentation may result from multiple factors, including occlusive wrapping, the type of garlic used (fresh or aged), duration of application, and other confounding metabolic variables. The use of a cotton bandage to secure the garlic to the skin may have exacerbated the burn by prolonging allicin’s contact with the tissue. Additionally, the use of freshly crushed garlic could have produced a higher-than-normal allicin concentration beneath the dressing. In combination, factors may account for the rapid onset of injury in our patient despite the significantly shorter length of exposure than previously reported. A higher concentration of allicin may also account for the deep tissue damage observed in the two larger bullae, rather than the more superficial vesiculobullous changes reported previously. The patient’s prior diagnosis of uncontrolled type 2 diabetes may have further heightened his susceptibility to a severe reaction.

The treatment approaches in these case reports were generally similar, involving topical antibiotic ointments and pain management; however, outcomes could not be compared due to a lack of patient follow-up [10-12].

## Conclusions

This case highlights the potential for atypical presentations and rapid onset of garlic-induced burns. Clinicians should be aware of the potential risks of at-home remedies and should be prepared to discuss alternative safe practices with their patients. We recommend that patients not attempt at-home remedies unless previously discussed with their healthcare provider. Specifically, regarding the use of garlic as an at-home remedy, it is safer for patients to utilize oral supplements or topical creams that are able to regulate the concentration of allicin. When a patient believes they have been exposed to a chemical burn, we recommend they rinse the exposed area with cold water for at least 30 minutes, remove contaminated items, and avoid immediate ointment application. We recommend that the patient seek emergency medical care when they experience large or deep burns, are unsure of the chemical involved, or have head, face, groin, or hand involvement. 
